# Study on Commercially Available Membranes for Alkaline Direct Ethanol Fuel Cells

**DOI:** 10.1021/acsomega.3c01564

**Published:** 2023-06-02

**Authors:** Michaela Roschger, Sigrid Wolf, Andreas Billiani, Kurt Mayer, Maša Hren, Selestina Gorgieva, Boštjan Genorio, Viktor Hacker

**Affiliations:** † Institute of Chemical Engineering and Environmental Technology, Graz University of Technology, Inffeldgasse 25/C, 8010 Graz, Austria; ‡ Faculty of Mechanical Engineering, University of Maribor, Smetanova Ulica 17, 2000 Maribor, Slovenia; § Faculty of Chemistry and Chemical Technology, University of Ljubljana, Večna Pot 113, 1000 Ljubljana, Slovenia

## Abstract

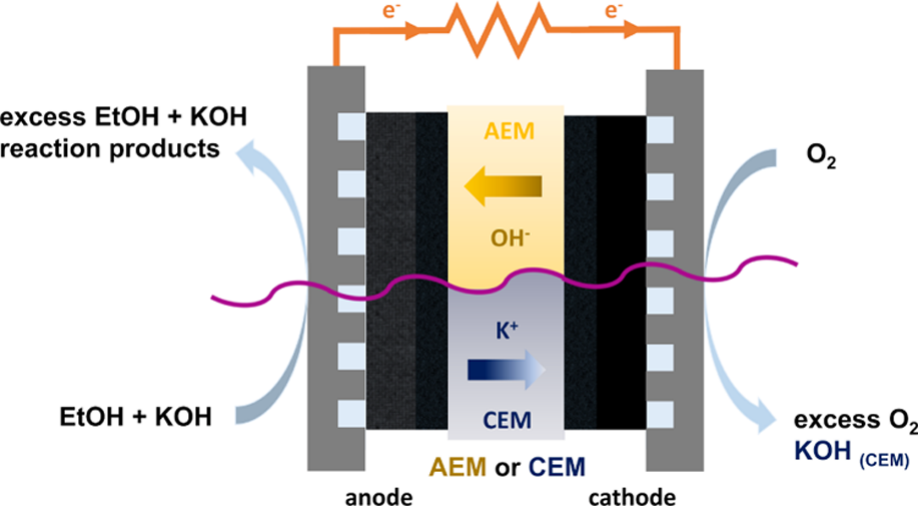

This study provides a comparison of different commercially available low-cost anion exchange membranes (AEMs), a microporous separator, a cation exchange membrane (CEM), and an anionic-treated CEM for their application in the liquid-feed alkaline direct ethanol fuel cell (ADEFC). Moreover, the effect on performance was evaluated taking two different modes of operation for the ADEFC, with AEM or CEM, into consideration. The membranes were compared with respect to their physical and chemical properties, such as thermal and chemical stability, ion-exchange capacity, ionic conductivity, and ethanol permeability. The influence of these factors on performance and resistance was determined by means of polarization curve and electrochemical impedance spectra (EIS) measurements in the ADEFC. In addition, the influence of two different commercial ionomers on the structure and transport properties of the catalyst layer and on the performance were analyzed with scanning electron microscopy, single cell tests, and EIS. The applicability barriers of the membranes were pointed out, and the ideal combinations of membrane and ionomer for the liquid-feed ADEFC achieved power densities of approximately 80 mW cm^–2^ at 80 °C.

## 1 Introduction

Fuel cells represent energy efficient devices for the production of electricity through electrochemical reactions. At the anode of the cell, the oxidation of the fuel takes place, and at the cathode, the reduction takes place. Generated electrons travel through an external circuit from the anode to the cathode and positive or negative ions, depending on the cell type, pass through the membrane to complete the circuit.^1−3^ In fuel cells where positive ions (cations) migrate, a cation exchange membrane (CEM) is used, while negative ions (anions) are migrating through an anion exchange membrane (AEM).^4^ Fuel cells that utilize AEMs belong to the category of alkaline fuel cells (AFC). AFCs have great advantages due to the caustic environment, such as better reaction kinetics and the possible use of non-precious metal-containing catalysts.^2,5−11^ Ethanol is one possible fuel for the AFC. It is carbon-neutral through its possible production from biomass, is easy to transport, and has a high energy density. Research on this alkaline direct ethanol fuel cell (ADEFC) has received an upturn due to its preferable properties compared to the toxic methanol. However, electrochemical cleavage is complex due to the C–C bond of ethanol with available catalysts. Complete conversion with OH^–^ ions, which are produced from the reduction of O_2_ at the cathode, results theoretically in the formation of CO_2_ and water at the anode.^2,4,6,9,12−15^ The transport of OH^–^ ions is achieved by the AEMs used, as already mentioned. However, these even commercial membranes from companies such as Solvay, Fumatech, Tokuyama, or Dioxide Materials face challenges such as durability, stability, and strength. Therefore, and due to the fact that the ethanol oxidation reaction is inhibited (C–C breaking issue), additional alkaline solution is added to the fuel in the ADEFC to provide enough OH^–^ ions for the reaction.^1,4,5,13,15,16^

**Figure 1:**
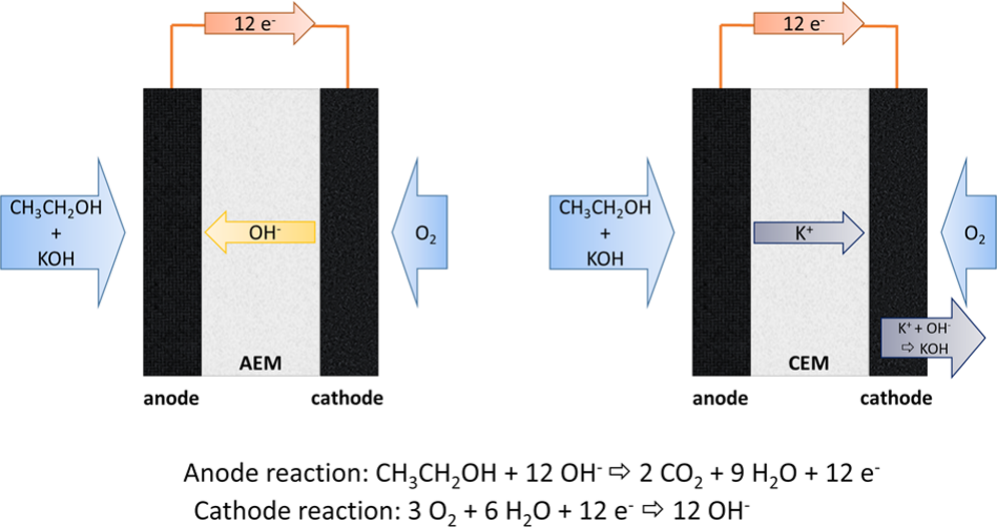
Schematics of transport processes and reactions in the ADEFC with an anion or cation exchange membrane (AEM or CEM).

Research on AEMs includes several strategies, such as the use of different manufacturing processes and the implementation of novel inorganic materials.^2,7,16−20^ Additional approaches address the use of a polymer, Nafion (designed by DuPont in 1962), which is used typically and with great success for the production of CEMs, due to the fact that the membranes have outstanding properties such as high stability, chemical resistance, and easy availability.^3,21−23^ These approaches include chemical modifications of the Nafion precursor by amination, or conversion of the membrane from a CEM to an AEM by anionic activation.^3,21,24−27^ They are based on the fact that the membrane is also used in the chlor-alkali industry and is therefore stable in high alkaline medium.^21^ An alternative option for the Nafion membrane in the ADEFC is to use it in its original application as a CEM to conduct K^+^ or Na^+^ ions to close the circuit.^8,28−31^ The two different operational modes of the ADEFC with AEM and CEM can be seen in Figure 1.

The electrochemical reactions at the anode and cathode are the same, but the migrating ion is different. This cell operation is enabled since KOH is added and thus OH^–^ is available for the ethanol oxidation reaction. In this case, since K^+^ (from the KOH) is conducted through the membrane, KOH is formed with the OH^–^ ions at the cathode in addition to the electrochemical reaction. However, this operation causes drawbacks, such as an increase in ethanol crossover, since the ions in this case do not migrate in the opposite direction to a possible ethanol diffusion (as is the case with OH^–^), and the generation of KOH can lead to corrosion problems and removal difficulties at the cathode.^8,28−31^

The same challenges which exist for AEMs also exist for anion exchange ionomers. Currently, anion exchange ionomers do not exhibit high chemical and thermal stability and ionic conductivity.^1,32−35^ Therefore, Nafion or the binder PTFE is in fact often used as well for the electrode production.^35^ Optimizations of electrodes with anionic ionomers and AEMs for AFC and AEM water electrolyzers, as well as the comparison with Nafion, can be found in the literature.^33,34,36−40^ The mixture of alkaline membranes and Nafion in the electrode was also employed and investigated in studies for alkaline direct fuel cells.^35,41−45^Table 1 shows a literature review of the use of selected commercial membranes and ionomers, but with various conditions (temperature, catalysts, and fuel) in the ADEFC.^8,18,21,25−29,31,41,42^

**Table 1:** Overview of the Literature of ADEFC Measurements with Commercial Selected Membranes and Ionomers

membrane	ionomer/binder	anode catalyst_(loading)_	cathode catalyst_(loading)_	anode fuel_(flow rate)_	cathode fuel_(flow rate)_	maximum power density_(T)_	refs
Nafion 211-Na^+^	PTFE	PdNi/C_(1 mg cm^–2^)_	Fe–Co K-14 HYPERMEC_(1 mg cm^–2^)_	3 M EtOH + 5 M NaOH_(2 mL min^–1^_)	oxygen_(100 sccm)_	100 mW cm^–2^_(60__°__C)_	(31)
						135 mW cm^–2^_(90__°__C)_	
Nafion 112-KOH		PtRu/C_(2 mg cm^–2^)_	Pt/C_(1 mg cm^–2^)_	2 M EtOH + 2 M KOH_(1 mL min^–1^)_	oxygen	33.65 mW cm^–2^_(60__°__C)_	(21)
						58.87 mW cm^–2^_(90__°__C)_	
Fumasep FAA3-PEEK	Nafion	PdAu/C_(1 mg cm^–2^)_	Pd/C_(1 mg cm^–2^)_	2 M EtOH + 2 M KOH_(1 mL min^–1^)_	oxygen_(500 mL min^–1^)_	44 mW cm^–2^_(85__°__C)_	(41)
Fumasep FAA3-PEEK	Nafion	PdSn/MWCNT_(1 mg cm^–2^)_	Pd/C_(1 mg cm^–2^)_	2 M EtOH + 2 M KOH_(1 mL min^–1^)_	oxygen_(500 mL min^–1^)_	18 mW cm^–2^_(60°C)_	(42)
						33 mW cm^–2^_(80°C)_	
						27 mW cm^–2^_(90°C)_	
Nafion 117-Na^+^	Nafion	PdNiSn/CF_(1 mg cm^–2^)_	Pt/C_(1 mg cm^–2^)_	2 M EtOH + 6 M NaOH_(3 mL min^–1^)_	oxygen	38.8 mW cm^–2^_(100°C)_	(8)
Nafion 117-OH^–^		PdNb/C_(1 mg cm^–2^)_	Pt/C_(1 mg cm^–2^)_	2 M EtOH + 1 M KOH_(1 mL min^–1^)_	oxygen_(200 mL min^–1^)_	27 mW cm^–2^_(60°C)_	(25)
Nafion 212-K^+^	PTFE/Nafion	Pd/C_(1 mg cm^–2^)_	Pt/C_(2 mg cm^–2^)_	3 M EtOH + 3 M KOH_(2 mL min^–1^)_	oxygen_(100 mL min^–1^)_	56.3 mW cm^–2^_(60°C)_	(28)
Nafion 117-OH^–^		Pd_10_(CeO_2_ NR)_20_(Vn)_70(1 mg cm^–2^)_	Pd_15_(CeO_2_ NR)_10_(Vn)_75(1 mg cm^–2^)_	2 M EtOH + 1 M KOH_(1 mL min^–1^)_	oxygen_(200 mL min^–1^)_	65 mW cm^–2^_(80°C)_	(26)
Nafion 117-OH^–^		Pd/C_(1 mg cm^–2^)_	(Pd_3_Pt_1_)_15_(CeO_2_ NR)_10_(Vn)_75(1 mg cm^–2^)_	2 M EtOH + 1 M KOH_(1 mL min^–1^)_	oxygen_(200 mL min^–1^)_	54 mW cm^–2^_(70°C)_	(27)
						61 mW cm^–2^_(80°C)_	
Fumasep FAA-3-50	Fumion FAA-3	PdNiBi/C_(0.75 mg cm^–2^)_	PtRu/C_(0.5 mg cm^–2^)_	3 M EtOH + 5 M KOH_(5 mL min^–1^)_	oxygen_(25 mL min^–1^)_	13.8 mW cm^–2^_(57°C)_	(18)
Nafion 212-K^+^		Pd/Ni foam_(0.35 mg cm^–2^)_	Pt/C_(2 mg cm^–2^)_	3 M EtOH + 3 M KOH_(2 mL min^–1^)_	oxygen_(100 mL min^–1^)_	30 mW cm^–2^_(60°C)_	(29)

Therefore, the aim of this study was to compare (same fuel, electrodes, and temperature) different low-cost AEMs from Fumasep (FAA-3-50, FAA-3-PK-75, and FAS-30) and a microporous separator FAAM-40 with a Nafion membrane that conducts K^+^ and an anionic-treated Nafion membrane for their use in the liquid-feed ADEFC. This study focuses on a set of selected membranes that are commercially available. The membranes were first investigated with respect to their physical and chemical properties (thermal and chemical stability, ion-exchange capacity, ionic conductivity, and ethanol permeability) which have an impact on cell performance and were finally studied in the single cell. In addition, the associated ionomers and their influence on the structure of the catalyst layer were analyzed. The limits of the membranes’ applicability were clearly shown and the optimal combination of membrane and ionomer for the ADEFC was declared.

## 2 Experimental Section

In this study, commercial membranes and ionomers were investigated for their use in the liquid-feed ADEFC; thus, all measurement methods and their performance have been carried out in harsh reaction conditions and with respect to the cell measurement and their proper application in the fuel cell.

### 2.1 Chemicals and Materials

Ultrapure water [∼18 MΩ cm, Barnstead NANOpure-Water Purification system (Dubuque, IA, USA)], sulfuric acid (H_2_SO_4_, 98% p.a., Chem-Lab NV, Zedelgem, Belgium), hydrogen peroxide (H_2_O_2_, TR 30%, BRENNTAG CEE GmbH, Wien, Austria), and potassium hydroxide (KOH, 0.1 M Fixanal 1 L Ampoule, Sigma-Aldrich, Darmstadt, Germany). KOH (≥85%, p.a., pellets), ethanol (EtOH, 99.9% p.a.), 2-propanol (99.9% p.a.), and hydrochloric acid (HCl, ROTIPURAN 37% fuming, p.a., ACS, ISO) were delivered by Carl Roth (Karlsruhe, Germany). Carbon paper (Sigracet 29 BC, 0.235 mm thick) and carbon cloth (ELAT-Hydrophilic Plain Cloth, 0.406 mm thick) and Fumion FAA-3 solution (10 wt % in NMP) were purchased from fuel cell store (College Station, TX, USA). Fumasep FAA-3-50 (anion-exchange membrane, non-reinforced, 50 μm), Fumasep FAA-3-PK-75 (anion-exchange membrane, PK-reinforced, 75 μm), Fumasep FAS-30 (anion-exchange membrane, non-reinforced, 30 μm), and Fumasep FAAM-40 (microporous separator, non-reinforced, 40 μm) were delivered by Fumatech (Bietigheim-Bissingen, Germany). Nafion Solution (NS-5, PFSA 5%) and Nafion 212 (cation-exchange membrane, 50 μm) were purchased from Quintech (Göppingen, Germany). A PdNiBi/C anode catalyst and a Ag–Mn_*x*_O_*y*_/C cathode catalyst from our previous literature were used.^46^

### 2.2 Activation-Treatment and Conditioning of the Membranes

The Fumasep FAA-3-50, FAA-3-PK-75, and FAS-30 membranes were supplied in the dry bromide form. Therefore, they had to be converted into the OH^–^ form by soaking them in 1 M KOH for 24 h and then cleaning them with ultrapure water.

The Fumasep FAAM-40 non-reinforced microporous separator has no functional groups for the transfer of the OH^–^ ions. Therefore, conditioning of the membrane sample was performed in 6 M KOH solution for 24 h.

The Nafion membrane (Nafion CEM) was used as it was received or treated with a procedure for anionic treatment (entitled Nafion AEM) as described in literature.^25^ The procedure is shortly described here: the membranes were first boiled in 1 M H_2_SO_4_ and then in 3 vol. % H_2_O_2_ solution at 80 °C for 1 h for each step. In between, they were washed with ultrapure water. Then, they were placed in 6 M KOH solution for 24 h. After extensive washing (three times with ultrapure water at 80 °C for 1 h each time), they were stored in 6 M KOH solution.

For comparison with the Fumasep membranes, the Nafion CEM was also immersed in 1 M KOH before the measurements and thus turned into an K^+^ conducting membrane.^28−30^

It is important to note here that the pretreatment of the Nafion membrane was done to convert it from a CEM to an AEM.^27^ The other membranes were conditioned for alkaline fuel cell application and the determination of ion exchange capacity and ionic conductivity.

### 2.3 Primary Properties of the Membranes

#### 2.3.1 Ion Exchange Capacity

Ion exchange capacity was measured by back titration of the membranes with 0.1 M KOH.^47^ For this purpose, they were immersed in 1 M KOH (FAA-3-50, FAA-3-PK-75, FAS-30, and Nafion CEM) or 6 M KOH (FAAM-40 and Nafion AEM), then in ultrapure water, and finally in 0.1 M HCl solution for 24 h in each respective solution. Titration was automated using a titrator (TitroLine 7800, SI Analytic, xylem, Washington D.C., USA). The calculation was performed using eq 1 (*V*_blank_: consumed KOH without sample, *V*_M_: consumed KOH with membrane, *c*_HCl_: concentration HCl, *w*_dry_: weight dry membrane).^7^
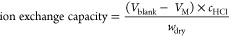
1

#### 2.3.2 Ionic Conductivity

Electrochemical impedance spectroscopy (EIS) was used to determine the ionic conductivity of the membranes.^47^ For this purpose, the membranes were immersed in 1 M KOH (FAA-3-50, FAA-3-PK-75, FAS-30, and Nafion CEM) or 6 M KOH (FAAM-40 and Nafion AEM) for 24 h and then in water for the same period of time. The measurement was performed with a Gamry Reference 600 potentiostat (GAMRY Instruments, Warminster, PA, USA) and a BekkTech BT110 LLC 205 (Scribner Associates, Southern pines, NC, USA) in a frequency range of 0.1 Hz to 10 kHz (amplitude: 50 mV) at 80 °C (initial start point for the stability determination). Equation 2 was used to determine the ionic conductivity (*R*_M_: membrane resistance, *d*: distance between electrode sense, *W*: width of membranes, and *T*: thickness of membranes).
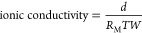
2

The real membrane resistance (*R*_M_) is obtained by determining the resistance of the membrane in ultrapure water (*R*_tot_: intersection with the *x*-axis of the Nyquist diagram) and afterward measuring only the ultrapure water with the same measuring principle (*R*_UPW_). *R*_M_ can then be calculated by eq 3.^48^
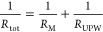
3

### 2.4 Thermal and Chemical Stability of the Membranes

#### 2.4.1 Thermal Stability

In order to determine the thermal stability of the membranes, thermogravimetric analysis with STA 449 C Jupiter apparatus from NETZSCH (Selb, Germany) was performed. The membranes were cut to a size of approximately 1 cm^2^ and then placed on a corundum plate sample holder. The measurements were performed in a temperature range from 30 to 900 °C with a heating rate of 10 K min^–1^ in a N_2_ atmosphere.

#### 2.4.2 Dimensional Changes in KOH

The dimensional changes [in-plane (eq 4) and through-plane (eq 5)] of the membranes were analyzed in 5 M KOH at 80 °C with the following time intervals: 1 and 24 h. For this purpose, their dimensions and thicknesses were measured before (*A*_dry_ and *T*_dry_) and after (*A*_wet_ and *T*_wet_) immersion in KOH.^16,47^
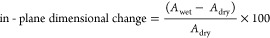
4
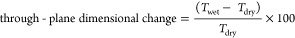
5

#### 2.4.3 Ethanol–Alkaline Stability Determined with Conductivity Measurements

The ethanol–alkaline stability of the membranes was determined by placing them in a mixture of 5 M KOH and 3 M EtOH solution at 80 °C and the ion conductivity measurements were carried out at specific time intervals (5, 24, 72, 144, and 288 h) after extensive washing with ultrapure water.^18^

### 2.5 Ethanol Fuel Permeability

The ethanol permeability through the membranes was measured using two diffusion cells at RT. These were filled on one side with 5 M KOH (reservoir B) and on the other side with a mixture of 5 M KOH and 3 M EtOH (reservoir A), respectively, with the membrane located at the center. The ethanol permeability in eq 6 (*c*_B0_: initial concentration of reservoir B, *c*_B_: concentration after the time interval, *t*: time interval, *V*_B_: volume of reservoir B, *L*: thickness of membrane, *A*: surface of membrane, and *c*_A0_: initial concentration of reservoir A) was determined by measuring the ethanol concentration with a conductometer after 1 and 24 h.^16^
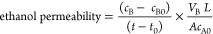
6

### 2.6 Single Cell Tests

The performance of the different commercial membranes and ionomers in the catalyst layer was investigated in a homemade ADEFC.^13^ A Ag–Mn_*x*_O_*y*_/C catalyst was used on the cathode side and a PdNiBi/C catalyst on the anode side, both on the support material Vulcan XC72R.^10,12,46^ These were processed into an ink using isopropanol, water, and ionomer (Nafion ionomer for all membranes or FAA-3 ionomer in addition for the FAA-3-50 membrane measurement) and sprayed onto the gas diffusion layer (GDL, anode: carbon cloth, cathode: carbon paper) using an ultrasonic spray coater (Sonotek, USA). An ideal active catalytic material loading of 0.5 mg cm^–2^ at the anode and 0.25 mg cm^–2^ at the cathode was achieved.^46^ The membranes were pretreated, by immersing in 1 M KOH (FAA-3-50, FAA-3-PK-75, FAS-30, Nafion CEM) or 6 M KOH (FAAM-40) for 24 h before the measurement (but not the Nafion AEM since it was already stored in 6 M KOH after the treatment). The membrane electrode assembly (MEA) was obtained by incorporating the two GDLs and the membrane together in the cell. Measurements were performed at room temperature (condition I), 60 °C (condition II), and 80 °C (condition III). The cell was operated with 5 M KOH and 3 M EtOH (5 mL min^–1^) at the anode and with pure (condition I) or humidified (condition II and III) oxygen (25 mL min^–1^) at the cathode. The polarization curves were recorded with a Zahner IM6ex potentiostat combined with a PP240 power potentiostat (Zahner-Elektrik GmbH & Co. KG, Kronach-Gundelsdorf, Germany).

#### 2.6.1 Electrochemical Impedance Spectra

The EIS were recorded between 50 kHz and 0.1 Hz at 440 mA (amplitude: 10%) under condition II. ZView software (Scribner Associates Inc., Southern Pines, NC, USA) was used for the evaluation and fitting (equivalent circuit model is shown in previous work^13^).

#### 2.6.2 Scanning Electron Microscopy

The morphology of the electrodes produced with different ionomers was investigated with scanning electron microscopy (SEM) with a Zeiss ULTRA plus. An aluminum SEM holder equipped with conductive carbon tape and SE2 and Inlens detectors at 2 kV or 5 kV at WD 5.5 mm were used.

## 3 Results and Discussion

The commercial membranes were first investigated for their physical and chemical properties (thermal and chemical stability, ion-exchange capacity, ionic conductivity, ethanol permeability) and then characterized in an ADEFC to examine their performance. The ex situ measurement methods were carried out with regard to the fuel cell conditions and were used to substantiate the performance of the membranes. Furthermore, the influence of two different ionomers on the electrode morphology and performance were investigated.

### 3.1 Ion Exchange Capacity and Ionic Conductivity

The ion exchange capacity and the ion conductivity are significant interrelated parameters that define the properties of the membranes and the performance inside the fuel cell. The ion exchange capacity specifies the quantity of ion-exchangeable groups, while the ionic conductivity describes the conductivity of ions through the membrane.^18^

**Figure 2:**
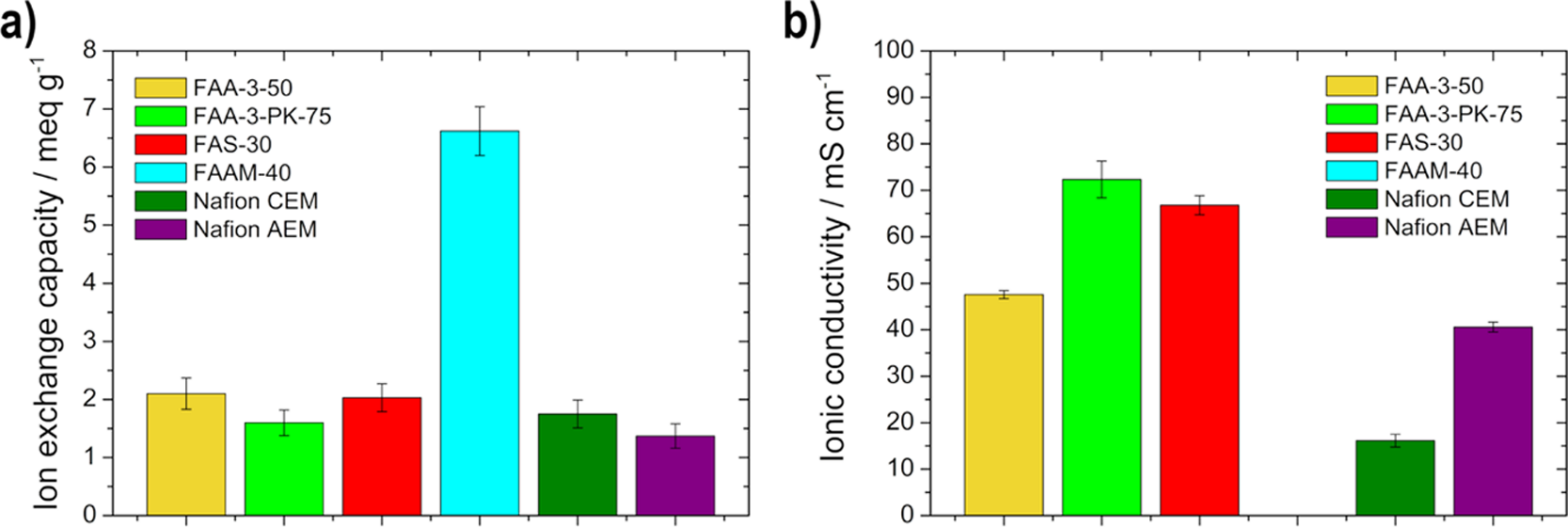
(a) Ion exchange capacity at RT and (b) ionic conductivity at 80 °C of the membranes (FAA-3-50, FAA-3-PK-75, FAS-30, FAAM-40, Nafion CEM, and Nafion AEM).

The IEC values, shown in Figure 2a, from the Fumasep membranes (FAA-3-50, FAA-3-PK-75, and FAS-30) are all in a similar range (2.1, 1.6, and 2.0 mequiv g^–1^), which is in agreement with the values available from the technical data sheet provided by the supplier. The IEC of the FAA-3-PK75 membrane is lower due to the reinforcement.^36^ The IEC values of Nafion are also slightly lower because the density of the polymer is higher.^36^ The anionic treatment and cleaning procedure resulted in a lower IEC for the Nafion AEM (1.4 mequiv g^–1^) than for the Nafion CEM (1.8 mequiv g^–1^). In the case of FAAM-40, which has no counterions, the exchangeable quantity of ions within the pores was most likely determined and is therefore exceptionally high (6.6 mequiv g^–1^).

The transport of OH^–^ ions through the membrane (ionic conductivity) can be defined by various transport mechanisms: Grotthuss mechanism, diffusion/migration, convection, surface hopping,^2^ whereby especially water molecules and clusters play an important role.^49^

The three Fumasep membranes (FAA-3-50, FAA-3-PK-75, and FAS-30) show higher conductivity values (Figure 2 b) compared to the others at 80 °C, with the following trend appearing: 48 mS cm^–1^ (FAA-3-50) < 67 mS cm^–1^ (FAS-30) < 72 mS cm^–1^ (FAA-3-PK-75). Khalid et al.^36^ noticed the same phenomena when measuring the conductivity of FAA-3-50 and FAA-3-PK-75 membranes at higher temperatures, due to the lower swelling of the FAA-3-PK-75 membrane. The quantity of mobile ions in the hydrophilic phase is reduced through excessive water absorption and thus counteracts the positive effect of crosslinking the hydrophilic domains and the conductivity.^2,36^ The conductivity of the untreated Nafion CEM (16 mS cm^–1^) is lower at 80 °C than that of the Nafion AEM (41 mS cm^–1^) because, on the one hand, it was only pre-conditioned in 1 M KOH instead of 6 M KOH^21^ and the transport of K^+^ is slower than the assumed transport of OH^–^ through anionic activation of the membrane. Yu et al.^30^ showed that Na^+^ conductivity is lower than H^+^ through Nafion, as well as An and Zhao^31^ showed that the transport of OH^–^ is easier through an AEM than Na^+^ through a CEM, and Hu et al.^23^ showed that the transport of Na^+^ ions is better than K^+^ ions in Nafion membranes treated with KOH and NaOH, although the conductivity of KOH is better than NaOH.^21^ Moreover, the ionic conductivity of protons is less dependent on temperature.^36^ The penetration of OH^–^ ions into the Nafion AEM is possible as a result of the swelling behavior (hydrophilic and hydrophobic regions) of the membrane in aqueous media. This results from the phase-separated structure of the membrane.^1,23^ In addition, Nafion exhibits higher water diffusion coefficients than FAA-3 due to morphological reasons.^49^ The FAAM-40 microporous separator exhibits no ionic conductivity since there are no functional groups for the conduction. The transfer of OH^–^ ions is only possible by using an electrolyte; however, as this conductivity measurement was carried out in water, this condition is not fulfilled.

### 3.2 Thermal and Chemical Stability of the Membranes

Thermal and chemical stability together with ion exchange capacity and ion conductivity are important requirements that membranes should meet in order to be considered for use in ADEFCs. Although the ADEFC is a low-temperature fuel cell, i.e., it is operated below 100 °C, the membranes should not decompose in this temperature range and remain form stable while still not compromising on conductivity. The temperature behavior of the membranes was investigated by TGA, the dimensional change of the membranes was analyzed in 5 M KOH at the highest operating temperature of the fuel cell of 80 °C, and the chemical stability was measured with conductivity measurements over a long period of time by storing the membranes in the operating medium (3 M KOH and 5 M KOH) at 80 °C.

#### 3.2.1 Thermal Stability

**Figure 3:**
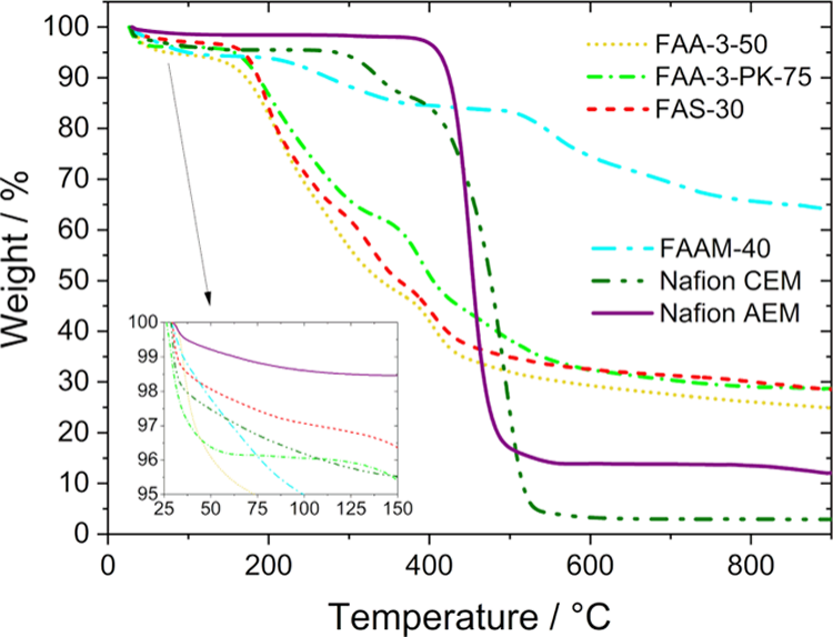
Thermogravimetric analysis curves of the membranes (FAA-3-50, FAA-3-PK-75, FAS-30, FAAM-40, Nafion CEM, and Nafion AEM) between 30 and 900 °C.

The thermal characteristics of all six membranes are shown in Figure 3. The three membranes from the company Fumatech (FAA-3-50, FAA-3-PK-75, and FAS-30) show a similar behavior since the thermal stability of AEMs is dependent on the polymer backbone.^2^ In the region from 30 to ∼150 °C the evaporation of water molecules from the membrane can be seen. At elevated temperatures, degradation and decomposition of the quaternary ammonium groups, the crosslinking bridges, as well as the polymer backbone, occur.^50^ In contrast, FAAM-40 is clearly most temperature stable, with 65% of the original weight remaining at 900 °C. However, the evaporation of water is also clearly visible and the degradation starts at ∼250 °C. The two Nafion variations CEM and AEM also undergo a weight loss as a result of hydration water. The weight reduction of pure Nafion membrane starting above 280 °C is due to desulfonation of the matrix and decomposition of the −SO_3_H group. At higher temperatures, the polar and nonpolar perfluorosulfonic vinyl ether and tetrafluoroethylene segments decompose, respectively. The lower stability of Nafion CEM compared to Nafion AEM results from the self-catalysis of the acid.^21,24^

#### 3.2.2 Dimensional Changes in KOH

**Figure 4:**
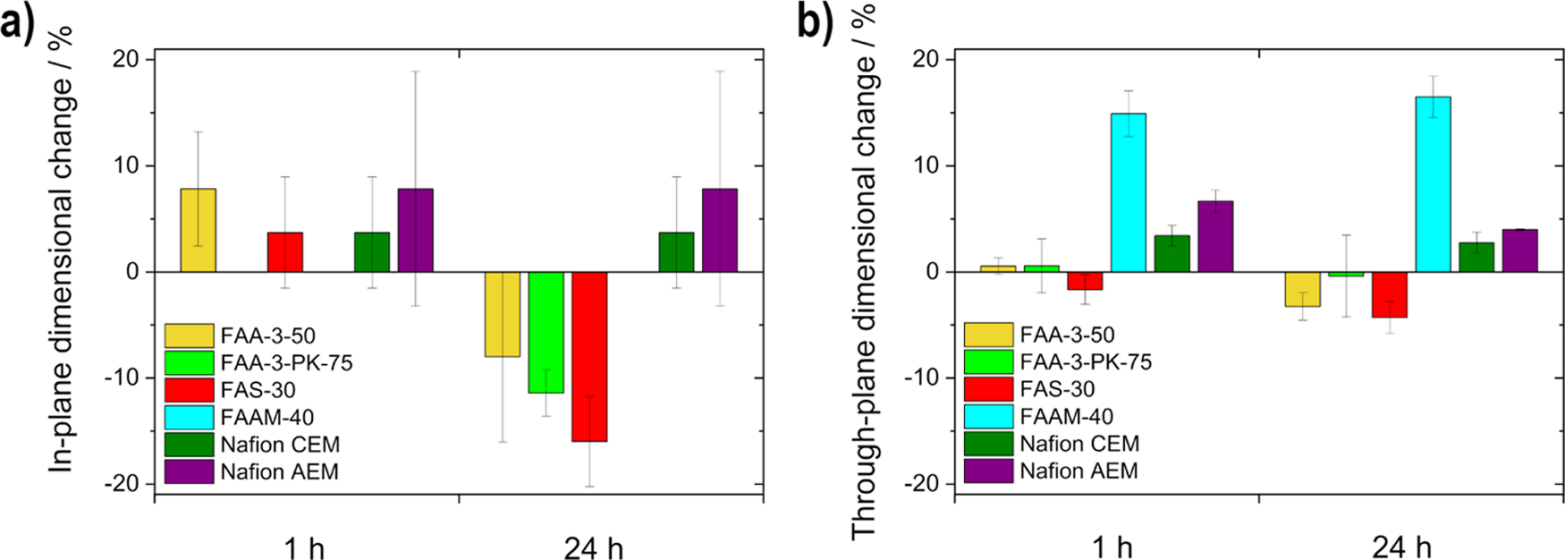
Dimensional changes (a) in-plane and (b) through-plane after 1 and 24 h of the membranes (FAA-3-50, FAA-3-PK-75, FAS-30, FAAM-40, Nafion CEM, and Nafion AEM) in 5 M KOH at 80 °C.

The dimensional change of the membranes was analyzed in 5 M KOH at 80 °C to obtain information about their chemical stability and swelling behavior after 1 and 24 h. The swelling behavior of the membrane in KOH, or its KOH uptake, has an influence on the performance and resistance within the cell since it affects the transport properties of the ions, as well as the contact to the electrodes.^3,16,19^Figure 4 shows the dimensional change for in-plane and through-plane.

The Fumasep (FAA-3-50, FAA-3-PK-75, and FAS-30) membranes show different behavior after 1 h than after 24 h. After 1 h, they tend to increase in size (FAA-3-50 and FAS-30) or remain the same (FAA-3-PK-75), which is due to the reinforcement of the FAA-3-PK-75 membrane (layered structure with support).^36^ After 24 h, however, they all decrease in size due to degradation. The high KOH (OH^–^) concentration and the high temperature enable degradation mechanisms on the AEMs such as the nucleophilic displacement reaction or Hoffmann elimination reaction.^1,21,31^ By contrast, there is scarcely any difference between the different time intervals for both Nafion membranes, due to the fact that Nafion membranes are robust even in strong alkaline conditions.^31^ The untreated Nafion CEM shows a lower swell behavior than the Nafion AEM. The swell behavior of Nafion in KOH is low, as shown by Hu et al.,^23^ so it can be concluded that the anionic treatment improved the KOH uptake. The microporous separator FAAM-40 is dimensionally stable even in highly concentrated KOH and elevated temperature, as no size loss is noticeable over time. However, swelling is only noticeable through-plane due to the filling of the pores.

#### 3.2.3 Ethanol–Alkaline Stability Determined with Conductivity Measurements

**Figure 5:**
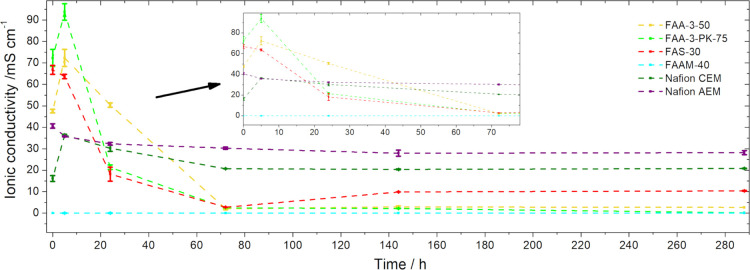
Ethanol–alkaline stability in 3 M EtOH and 5 M KOH solution at 80 °C measured with ionic conductivity of the membranes (FAA-3-50, FAA-3-PK-75, FAS-30, FAAM-40, Nafion CEM, and Nafion AEM).

In addition to the dimensional change measurement of the membranes in 5 M KOH at 80 °C, the change in their ionic conductivity (Figure 5) was investigated at different time intervals by placing them in 5 M KOH and 3 M EtOH at 80 °C. This measurement principle provides information on the duration of the membrane’s conductivity under the highest operating conditions and thus its operational lifetime.

All of the Fumasep membranes (FAA-3-50, FAA-3-PK-75, and FAS-30) show significant and rapid degradation, as almost no conductivity is measurable after 72 h. The conductivity loss of the Fumasep membranes (FAA-3-50, FAA-3-PK-75, and FAS-30) is due to the degradation of the cationic groups by attack of the hydroxide ions, as the conductivity correlates with the quantity of functionalized cationic groups.^1,9^ Khalid et al.^36^ showed that the FAA-3-50 membranes resistance increased drastically and thus the conductivity decreased due to the degradation of the quaternary ammonium groups through the reaction with OH^–^ ions. The minimal increase for the FAS-30 may be due to any remaining KOH in the membrane, as Liao et al.^19^ have shown that a higher conductivity is measured if the membrane is not cleaned before measurement. The increase in conductivity after 5 h for the FAA-3-50 and the FAA-3-PK-75 membrane is due to the higher concentration of KOH and the fact that possibly not all of the counter ion Br^–^ was replaced by OH^–^ during the pretreatment of the membranes.^18^ The Nafion AEM displays a very stable behavior over the entire measured time range. The first drop after 5 h is due to the fact that the initial value of the membrane was measured after storage in 6 M KOH, as mentioned above. The Nafion AEM delivers a good conductivity even after 288 h, which indicates a high stability. The similar observation is valid for the Nafion CEM, which however shows a short increase after 5 h due to the higher KOH concentration (more K^+^) than before (1 M KOH). The FAAM-40 microporous separator shows no conductivity over the entire measured range, which is due to the absence of functional groups for the transport of OH^–^, as already mentioned.

### 3.3 Ethanol Fuel Permeability

**Figure 6:**
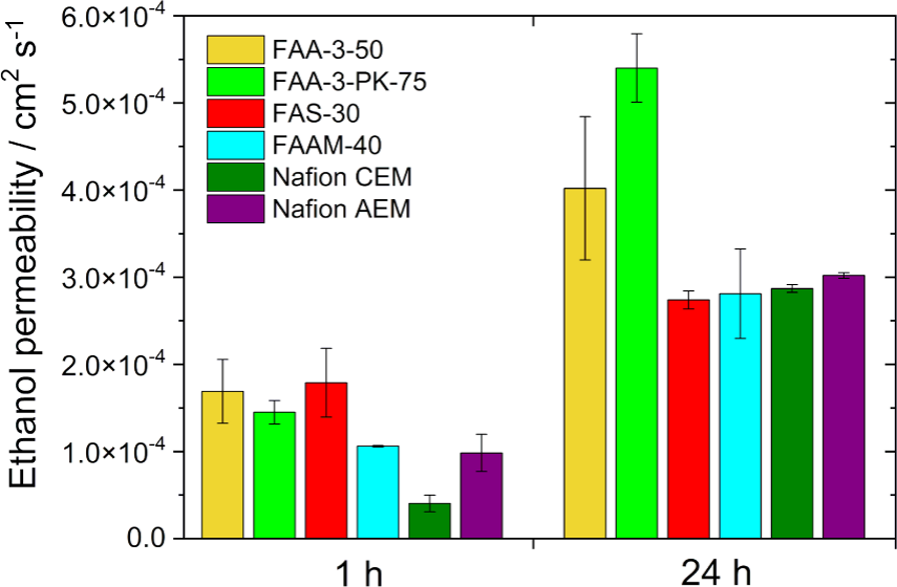
Ethanol permeability after 1 and 24 h of the membranes (FAA-3-50, FAA-3-PK-75, FAS-30, FAAM-40, Nafion CEM, and Nafion AEM) at 25 °C.

In the operation of the ADEFC, ethanol permeability through the membrane plays an important role. Ethanol crossover through the membrane leads to a decrease in cell performance due to fuel loss at the anode and by interference with the cathode reaction to mixed-potentials.^10,11,16,22^ Therefore, the ethanol permeability through the membranes of 3 M EtOH in 5 M KOH was investigated after 1 and 24 h outside of the fuel cell with two diffusion cells. The results are shown in Figure 6.

The ethanol permeability increases with time for all membranes tested. The Nafion CEM showed the lowest ethanol permeability after 1 h, whereas the Fumasep membranes (FAA-3-50, FAA-3-PK-75, and FAS-30) exhibited the highest values. The ethanol permeability for the Nafion AEM and the FAAM-40 microporous separator are in the same range. After 24 h, FAS-30, FAAM-40, Nafion CEM, and Nafion AEM exhibit similar ethanol permeability values. However, the ethanol permeability of the FAA-3-50 membrane increases dramatically, and increases even more for the reinforced FAA-3-PK-75 membrane. The ethanol permeability is related to the through-plane swelling of the membranes. If the membrane swells over time, the ethanol permeability can be lower. The thickness of the FAA-3-PK-75 membrane does not really change due to its reinforcement and thus shows a higher ethanol permeability. Moreover, the alcohol crossover through Nafion membranes causes the membrane to swell and modifies the surface (more flat).^51^ With rising ethanol concentration, the membrane porosity increases and the membrane swells more; however, this is a reversible process as shown by Song et al.^52^ Kontou et al.^53^ observed that ethanol crossover is highly dependent on ethanol concentration and swelling behavior of the membrane due to structural changes occurring in the fluorocarbon matrix. This in turn explains the lower ethanol permeability of the Nafion membrane in comparison to the FAA-3-PK-75 membrane.

### 3.4 Performance Tests

The performance of the commercial membranes was investigated with single cell tests (polarization curves and EIS) in an ADEFC under the same conditions for all membranes (fuel, electrodes, and temperature). Furthermore, the influence of two different ionomers (FAA-3 and Nafion) on the electrode morphology and the performance was determined.

#### 3.4.1 Influence of the Membrane

**Figure 7:**
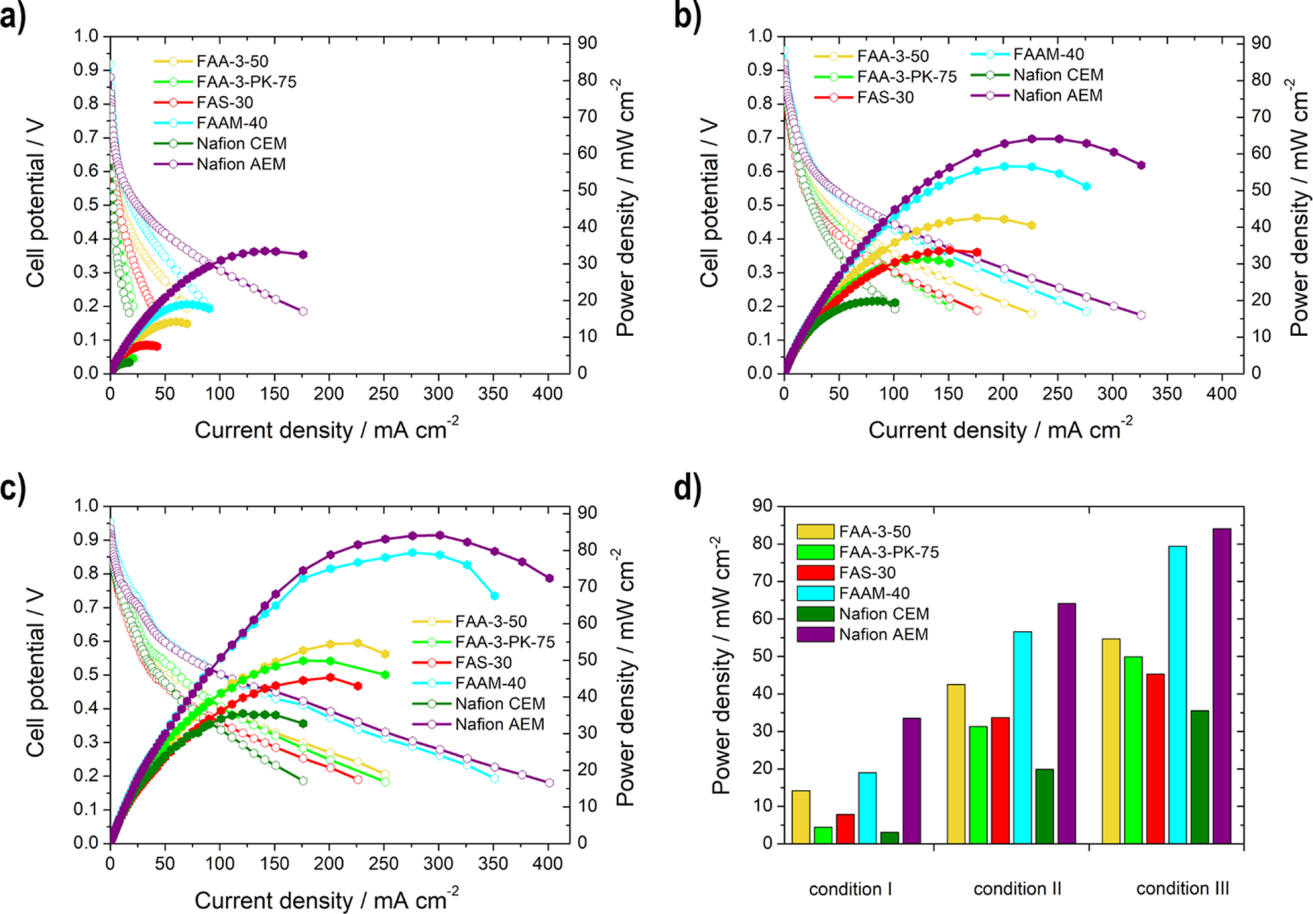
Single cell measurement results for the comparison of the membranes (FAA-3-50, FAA-3-PK-75, FAS-30, FAAM-40, Nafion CEM, and Nafion AEM): power density (filled symbols) and polarization curves (unfilled symbols) for (a) condition I, (b) condition II, and (c) condition III; (d) maximum power density for the different conditions.

The single cell measurements were performed at different temperatures (condition I: RT, condition II: 60 °C, and condition III: 80 °C) to show the effect of temperature on the membrane and the performance. The measurements were conducted with 3 M EtOH and 5 M KOH fuel at the anode side since higher performance can be achieved with this concentration according to Abdullah et al.^54^ All membrane measurements (Figure 7) show an increase in power density as the temperature rises, as a result of the increasing conductivity of the membrane, electrode kinetics, and mass transfer properties.^46^

The ranking of the membranes based on their maximum power density values is the same for all temperatures: Nafion CEM < FAA-3-PK-75 < FAS-30 < FAA-3-50 < FAAM-40 < Nafion AEM. However, there is one exception: at condition III, FAS-30 and FAA-3-PK-75 replace position, which is due to the higher conductivity of the FAA-3-PK-75 at 80 °C, as previously determined, and due to the reinforcement and the resulting higher stability of the membrane. The maximum power density values of the Nafion CEM (Table 2) are always the lowest among all membranes due to the different mode of operation of the cell. The K^+^ ions are transported from the anode to the cathode and only the added OH^–^ of the KOH can be used for the ethanol oxidation reaction. The AEMs from Fumatech are clearly preferred since they conduct additional OH^–^ from the cathode to the anode.^31^ The inferior performance of these membranes (FAA-3-50, FAA-3-PK-75, and FAS-30) compared to the FAAM-40 and Nafion is due to their lower stability and their use beyond their designated application range. Therefore, the highest performances can be achieved with the microporous separator FAAM-40 and with the Nafion AEM, which is due to their outstanding stability at high temperatures and highly alkaline conditions. It is important to note here that these observations are only valid for the case of this fuel combination. According to the technical data sheet provided by the supplier, the use of FAAM-40 would not be possible at lower KOH concentrations.

**Table 2:** Maximum Power Density and Open Circuit Voltage Values of the Single Cell Tests of All Membranes for Conditions I–III

	power density/mW cm^–2^			open circuit voltage/V		
condition	I	II	III	I	II	III
FAA-3-50	14.2	42.5	54.7	0.770	0.907	0.936
FAA-3-PK75	4.45	31.3	49.9	0.763	0.889	0.927
FAS-30	7.84	33.7	45.3	0.771	0.909	0.919
FAAM-40	19.0	56.6	79.4	0.918	0.958	0.952
Nafion CEM	3.14	19.9	35.5	0.821	0.905	0.901
Nafion AEM	33.5	64.1	84.1	0.880	0.920	0.934

The open circuit values of the single-cell measurements are reflected in Table 2 and may provide an indication of the occurrence of ethanol crossover, which is facilitated at higher temperatures through the membrane and consequently, of mixed potentials. In the case considered here, however, the possibility of mixed potentials forming is lower because an ethanol-tolerant catalyst was used.^10,46^ The values of the Nafion AEM compared to the Nafion CEM are always slightly higher, which is due to the fact that the presumed flux of ions (K^+^) is not directed against a possible crossover of ethanol when applying the Nafion CEM, but in the same direction. Thus, the electroosmotic drag is not reversed to the ethanol crossover, which supports the latter.^8^ The microporous separator FAAM-40 exhibits the highest OCV values. We assume that this is due to the excellent conduction of OH^–^ through the pores. Furthermore, the previously measured high ethanol permeability of the Fumasep membranes indicates the low OCV values at RT.

**Figure 8:**
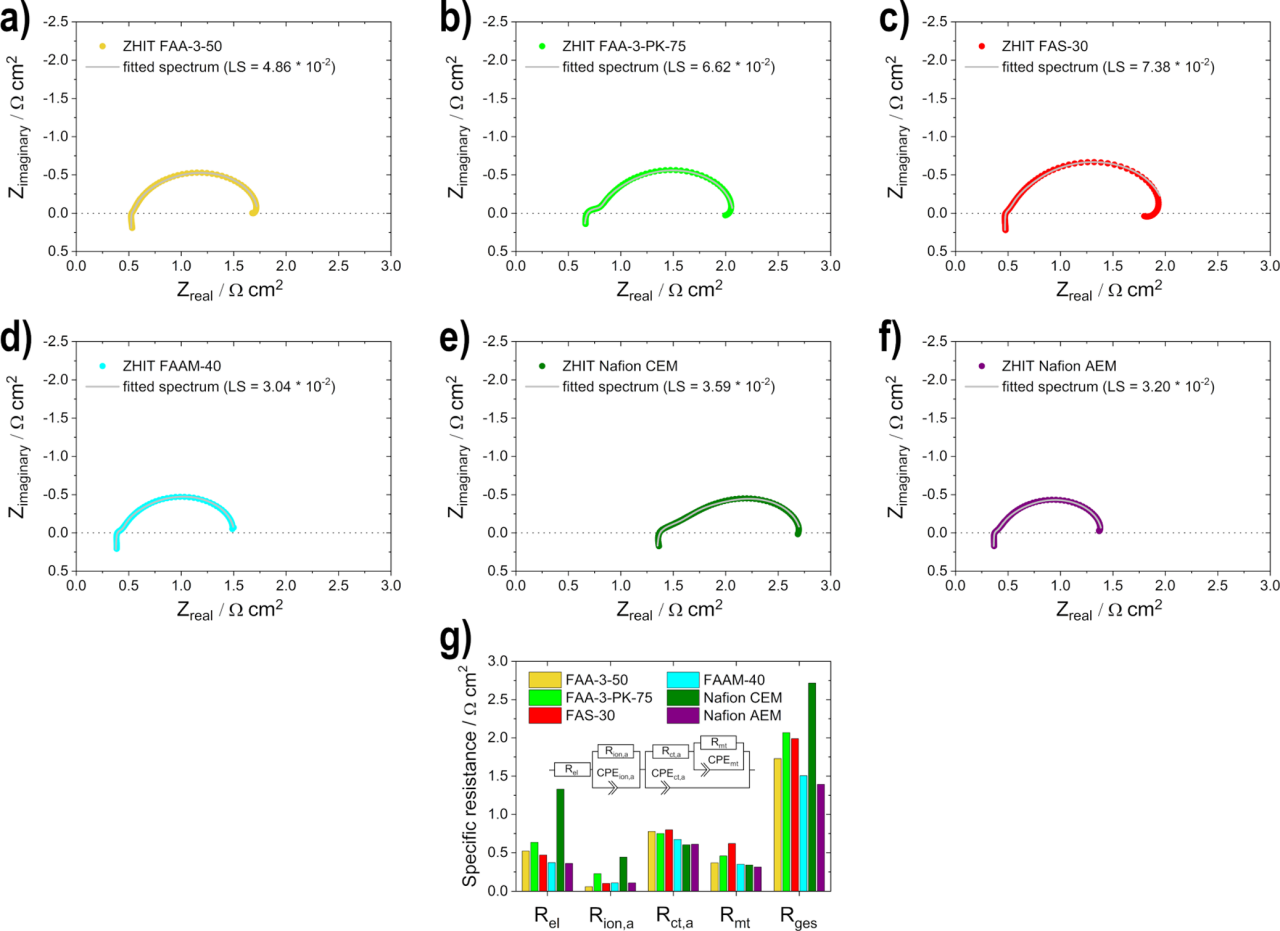
Electrochemical impedance spectra measurements (ZHIT algorithm) of the membrane measurements: (a) FAA-3-50, (b) FAA-3-PK-75, (c) FAS-30, (d) FAAM-40, (e) Nafion CEM, and (f) Nafion AEM and (g) comparison of the resistances at condition II [the shown inserted equivalent circuit is redrawn (for easier readability) from previous work:^13^ Roschger, M.; Wolf, S.; Mayer, K.; Singer, M.; Hacker, V. Alkaline Direct Ethanol Fuel Cell: Effect of the Anode Flow Field Design and the Setup Parameters on Performance. *Energies***2022, 15 (19),** 7234. https://doi.org/https://doi.org/10.3390/en15197234 which was published under a CC BY 4.0 license (https://creativecommons.org/licenses/by/4.0/). Copyright 2022 by the authors. License MDPI, Basel, Switzerland].

For better demonstration of the losses of the individual measurements with the different membranes, EIS was additionally carried out (Figure 8).

The overall resistance *R*_ges_ of the different measurements is for each individual in agreement with the measured maximum power density values of the same condition (condition II), meaning that if the resistance is high, the maximum power density is low and vice versa. Therefore, low overall resistances enable better cell performance. Whereby, the behavior between the membranes of *R*_ges_ is similar to the behavior of the electrolyte resistance *R*_el_ as well as the anodic ionomer resistance *R*_ion,*a*_. This is due to the fact that the same electrodes were used for the measurements and only the membrane was varied, causing these resistances (ion conductivity in different regions of the cell) to variate more than the anodic charge transfer resistance *R*_ct,*a*_ and the mass transfer resistance *R*_mt_.^46^ The Nafion CEM shows the highest *R*_ges_, *R*_el_, and *R*_ion,*a*_, due to the presumed slower transport (lower conductivity) of K^+^ than OH^–^, as shown before with the ex situ conductivity measurements. The Nafion AEM measurement resistance values are always the lowest and quite similar to the values of the FAAM-40; thus, the results support the fact that the anionic activation of the Nafion membrane in an AEM enables OH^–^ ion transport. The *R*_el_ values of the Fumasep membranes (FAA-3-50, FAA-3-PK-75, and FAS-30) are higher than the values of the Nafion AEM and also the FAAM-40. Conclusively, the influence of the membranes on the cell performance in relation to the resistance could be clearly determined.

#### 3.4.2 Influence of the Ionomer

A polymeric binder or ionomer is essential in the manufacture of electrodes in order to form a porous catalyst layer, bind the catalyst particles, and allow the transfer of reactants, products, ions, and electrons.^5,37^ The thermal and chemical stability of anion exchange ionomers, however, is currently still low.^5,9^ The stability of the ionomer, though, determines the overall stability of the electrode structure,^35^ for which the stable Nafion is often used.^34^ The influence of the ionomer (FAA-3 vs Nafion) with the same *I*/*C* ratio on the performance and properties of the electrode is discussed in the next section using a Fumasep FAA-3-50 membrane. The application of the Nafion ionomer is possible because the fuel contains enough OH^–^ (5 M KOH) for the supply of the three-phase boundary,^44^ or a contribution to the exchange of OH^–^ in the catalyst layer in alkaline cells could even be suggested.^35^

**Figure 9:**
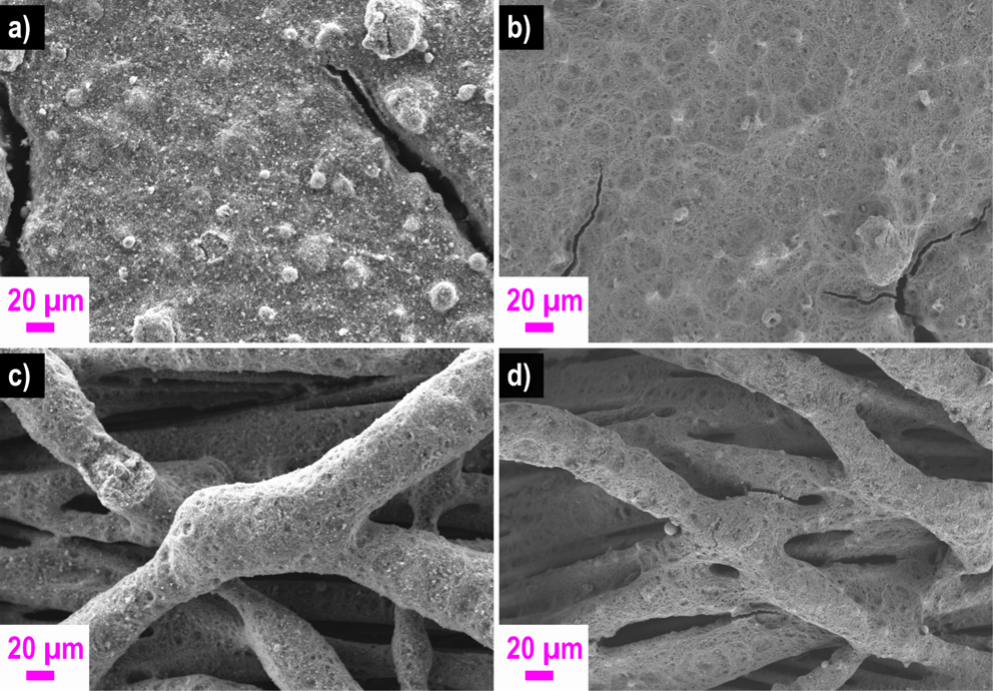
Morphology investigation of the electrodes produced using two different ionomers [FAA-3 (a,c) and Nafion (b,d)] with scanning electron microscopy: (a,b) cathode with Ag–Mn_*x*_O_*y*_/C and (c,d) anode with PdNiBi/C.

A clear difference in the structure and composition of the electrodes can be seen from the SEM images, but the behavior is independent of the gas diffusion layer used. In Figure 9a,c, the electrodes with FAA-3 ionomer and in Figure 9b,d with Nafion ionomer are shown. The electrodes made with FAA-3 ionomer show a film-like bonded structure and large agglomerates. By contrast, the electrodes prepared with the Nafion ionomer show a porous structure. The nature of the electrodes can be attributed to the influence of the ionomer during ink production. The ionomer has a great influence on the agglomeration and the stability of the dispersion. Film-like structures of the electrodes indicate agglomeration of the solution. However, a good pore structure is essential for the mass transport of the reactants, and moreover, the ion conductive path depends on the structure.^37,45^

**Figure 10:**
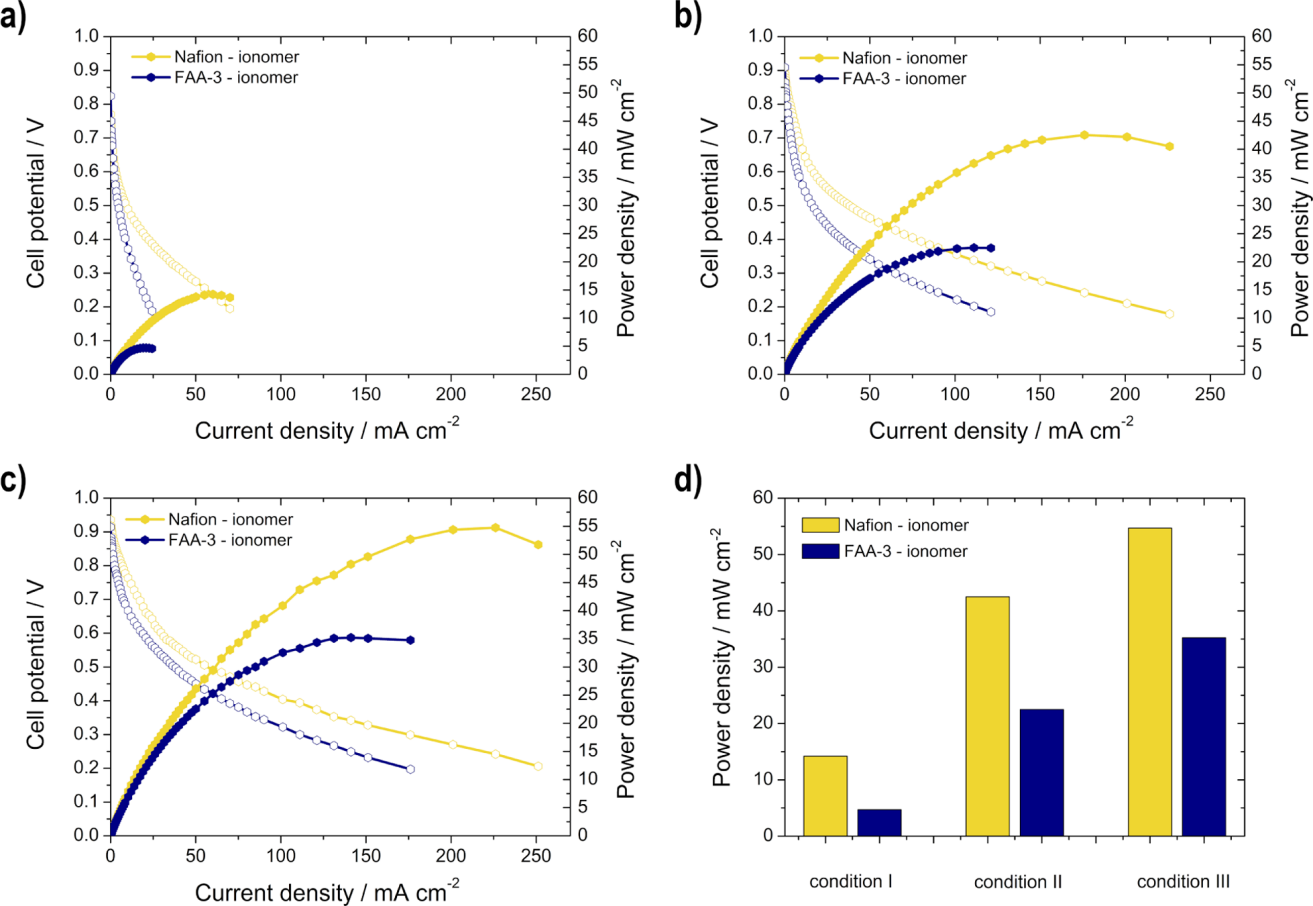
Single cell measurement results for the comparison of the two different ionomers (FAA-3 and Nafion ionomer): power density (filled symbols) and polarization curves (unfilled symbols) for (a) condition I, (b) condition II, and (c) condition III; (d) maximum power density for the different conditions.

The single cell measurements (Figure 10) of the different electrodes always show the same behavior independent of temperature: using the Nafion ionomer, higher power density values and thus better performance is achieved than with the FAA-3 ionomer [14.2 vs 4.71 mW cm^–2^ (condition I), 42.5 vs 22.5 mW cm^–2^ (condition II), 54.7 vs 35.2 mW cm^–2^ (condition III)].

This can be attributed to the porosity as previously determined with the SEM measurements and the influence of the ionomer on the accessibility of active sites. Roca-Ayats et al.,^34^ noted that the accessibility of active sites changes depending on the ionomer used: the same Fumion quantity in the ink causes more surface blockage than the same Nafion quantity. Moreover, they demonstrated that alkaline ionomers block active platinum sites and in particular low coordinated ones, which is due to the presence of bromide in the ionomer solution (strong adsorption on platinum surfaces).^34^ The appropriate correct content for the FAA-3 ionomer has not yet been investigated to the same extent as for the Nafion ionomer. However, there are a few studies available for the AFC. Carmo et al.^33^ showed that the optimal FAA-3 ionomer content is 25%, and that an excess (45%) leads to a decrease in catalyst utilization as gas penetration is blocked. Whereas Sebastián et al.^39^ pointed out that the optimum content is 50 wt % while replacing the Br^–^ with OH^–^ in the ionomer of the electrode. Kim et al.^40^ determined that an *I*/*C* value of 0.5 for both electrodes is ideal: excessive amount of ionomer blocks the active sites and the transport of gases and water, whereas an insufficient quantity restricts the movement of both ions and water.

**Figure 11:**
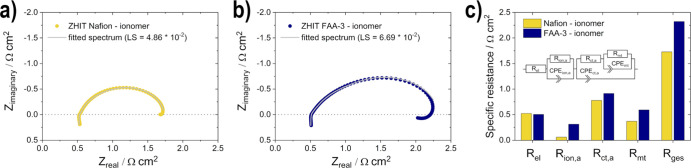
Electrochemical impedance spectra measurements (ZHIT algorithm) for the variation of ionomer: (a) Nafion-ionomer, (b) FAA-3-ionomer, and (c) comparison of the resistances under condition II [the shown inserted equivalent circuit is redrawn (for easier readability) from previous work:^13^ Roschger, M.; Wolf, S.; Mayer, K.; Singer, M.; Hacker, V. Alkaline Direct Ethanol Fuel Cell: Effect of the Anode Flow Field Design and the Setup Parameters on Performance. *Energies***2022, 15 (19),** 7234. https://doi.org/https://doi.org/10.3390/en15197234 which was published under a CC BY 4.0 license (https://creativecommons.org/licenses/by/4.0/). Copyright 2022 by the authors. License MDPI, Basel, Switzerland].

The EIS measurements in Figure 11 show the influence of the ionomer on the transport of reactants, products, ions, and electrons inside the fuel cell components. *R*_el_ is similar for both measurements since the same membrane was used. The overall resistance *R*_ges_ of the system is higher when the FAA-3 ionomer is used for the measurement since each *R*_ion,*a*_, *R*_ct,*a*_, and *R*_mt_ are increased. In addition, the inductive loop in the low-frequency region, which results from adsorbed intermediate species on the EOR catalyst, is enlarged.^55^ These observations are consistent with the previously described phenomena, namely, the reduced porosity of the electrode and the accessibility of the active sites.

These results show the significant influence of the electrode structure on the mass transport and ion conduction and also the effect of the ionomer on catalytic activities and thus represent a major impact on the cell performance.

## 4 Conclusions

Different low-cost AEMs (Fumasep FAA-3-50, FAA-3-PK-75, FAS-30), a microporous separator (Fumasep FAAM-40), a CEM (Nafion), and an anionic treated CEM (Nafion) were compared in the liquid-feed ADEFC with the same fuel, electrodes, and temperatures. In addition, the influence of the associated ionomers (FAA-3 and Nafion) on the electrode porosity and activity was analyzed. The ex situ analyses (thermal and chemical stability, ion-exchange capacity, ionic conductivity, ethanol permeability) of the membranes were carried out with respect to their application in the fuel cell. The Fumasep membranes showed high ion conductivity and good ion exchange capacity values in slightly alkaline conditions, but poor thermal stability, as well as poor chemical stability and increased ethanol permeability in the highly basic fuel. In comparison, the anionic-treated Nafion membrane and the FAAM-40 microporous separator were outstanding for their high alkaline stability and low ethanol permeability. The drawbacks of the operation of the ADEFC with the CEM could be pointed out, which are a higher ethanol crossover rate and the lower K^+^ conductivity.

Furthermore, the ionomer-type effects the electrode structure and the catalytic activities, with regard to transport and conduction properties, and thus, the power output of the fuel cell. Higher performance was achieved with the Nafion ionomer than with the FAA-3 ionomer. The interaction of all the mentioned properties resulted in the highest maximum power densities of 79.4 and 84.1 mW cm^–2^ at 80 °C with the use of the FAAM-40 microporous separator and the anionic-treated Nafion membrane, respectively.

The study was thus able to identify and evaluate the limits of applicability of the available membranes and ionomers in the ADEFC as a basis for further research and performance improvement.
